# Using Pen-Side Measurable Blood Parameters to Predict or Identify Dystocic Lambing Events

**DOI:** 10.3390/biology11020206

**Published:** 2022-01-27

**Authors:** Amellia Redfearn, Jody McNally, Heather Brewer, Emma Doyle, Sabine Schmoelzl

**Affiliations:** 1 FD McMaster Laboratory, Agriculture and Food, Commonwealth Scientific and Industrial Research Organisation, Armidale, NSW 2350, Australia; amellia.redfearn@csiro.au (A.R.); jody.mcnally@csiro.au (J.M.); heather.brewer@csiro.au (H.B.); 2 School of Environmental and Rural Science, University of New England, Armidale, NSW 2351, Australia; edoyle3@une.edu.au

**Keywords:** sheep, ewe, reproduction, production, physiology, rapid testing, time course

## Abstract

**Simple Summary:**

Prolonged or non-progressive labour is the greatest risk factor for loss of newborn lambs in Australia and poses significant welfare and economic concerns worldwide. In this study, we set out to investigate whether pen-side technology could be used to predict which ewes would be at risk of prolonged labour. In our pilot trial, we found potentially useful markers. We next developed a sampling protocol by looking at changes in candidate markers over time in normal lambing events. Finally, we searched for blood markers that could distinguish between normal and difficult lambing events, sampling pre-birth (estimated one week before birth), at birth (within 3 h) and post-birth (16–26 h). Possible predictors of lambing difficulty were chloride, haematocrit and haemoglobin, sampled one week before birth; creatinine, sampled at birth; and acid–base related parameters after birth. In conclusion, we found that pen-side analysis of blood markers showed promise in identifying dystocic lambing events. More information is required to decide whether pen-side diagnostics could be useful to identify and predict dystocic lambing in the future.

**Abstract:**

Dystocia is the greatest contributor to neonatal lamb mortality in Australia and poses significant welfare and economic concerns worldwide. In this study, we set out to investigate whether pen-side analysis technology could be employed to detect blood parameters predictive of dystocic labour events in sheep. In a pilot trial, we collected and analysed blood samples in pen-side assays for glucose, lactate, pH, pCO_2_, pO_2_, base excess, HCO_3_, TCO_2_, sO_2_, lactate, sodium, potassium, chloride, calcium, urea nitrogen, creatinine, haematocrit, haemoglobin and anion gap. From the pilot data, we identified creatinine, TCO_2_, chloride and calcium as potentially useful markers. To develop a time course and to establish variability of the selected blood parameters, a time series of samples was collected from 12 ewes, from mid-gestation to 48 h after birth. For the main trial, blood samples were collected at mid- and late gestation for glucose determination and for the full set of blood parameters at three time points before, at and after birth. Possible predictors of lambing difficulty were chloride, haematocrit and haemoglobin, sampled one week before birth; creatinine, sampled at birth; and blood pH and base excess after birth. In conclusion, we found that pen-side analysis of blood markers showed promise in identifying dystocic lambing events.

## 1. Introduction

Dystocia is an important welfare and economic issue faced in animal production industries in Australia and across the world. It is the greatest contributor to neonatal lamb mortality [1] and lamb and ewe morbidity in Australia [2]. Over many years of research, lamb mortality rates have remained troublingly high, as described by [1,3], irrespective of increased lambing rates, driven by increased average litter size. Much of the existing body of research investigates the effects of management [4,5] and genetic factors [6,7] on lamb mortality. Lamb loss is the end point of a multifactorial complex process with considerable influence of uncontrollable environmental factors, such as prevailing weather around the time of lambing, which means research data on lamb survival tend to be highly variable, making research project effortful and progress slow. In our research, we focused on better understanding the underlying causes of dystocia as the main risk factor leading to lamb loss [1]. 

Blood biochemistry analyses are able to provide insights into individual animals’ health and welfare status by identifying disease presence, metabolic disturbances and stress hormone levels [8,9]. Traditionally, samples are collected, stored and processed in a laboratory. These tests can be costly and may take days to process, especially for large sample sizes. It has been reported that the type of collection tube used, temperature at which samples are stored, the length of time samples are stored and the number of times samples are frozen and thawed significantly impact the results received [9]. Therefore, the development of point-of-care analysis technology has introduced fast, portable, affordable and easy-to-use laboratory-equivalent tests to veterinary and research settings [10,11]. These technologies have made multifactorial health and welfare investigations more easily carried out on an individual and group basis.

In this study, we made use of a series of lambing trials designed to collect behavioural data during lambing [12] to collect blood samples and obtain information on maternal blood parameters. In an initial pilot trial, samples were collected immediately after birth and 24 h after birth. This was followed by a time course trial to refine our sampling protocol. Finally, in the main trial phase, pre- and post-birth samples were collected, endeavouring to identify differences in the blood profiles between eutocic and dystocic lambing events.

## 2. Materials and Methods

Animal experimentation was undertaken at the CSIRO FD McMaster Laboratory, Armidale, NSW, Australia between March 2018 and November 2019 and conducted in accordance with the Australian Code for the Use and Care of Animals in Research and Teaching. Experimental protocols were approved by the CSIRO Armidale Animal Ethics Committee under Animal Ethics Approval numbers 18/11 and 19/10.

### 2.1. Animal Management

All animals were treated in the same manner and followed the same lambing protocols unless stated otherwise. All trials were made up of pure Merino ewes of varying ages (2–7 years old), mated to either Merino or Border Leicester rams, and carrying either singles or twins. Each trial was balanced as far as possible for even numbers of these combinations. 

Approximately one week before lambing was due to commence, ewes were brought into the yards. The 20 ewes that were due to lamb first were drafted and were assigned to group 1, and the rest of the ewes were divided evenly into groups based on due date. All ewes were side branded with a unique number with stock safe paint (Siromark, Heininger, Bibra Lake, WA, Australia) to facilitate identification from video records. Group 1 was moved into the supervised lambing enclosure equipped with 10 day- and night-vision cameras (Hikvision Digital Technology Co, Hangzhou, China) recording continuously for the entire trial period, and the remaining groups were held in nearby holding paddocks. Ewes had ad libitum access to water and pasture grass and were supplemented with a mix of Faba beans and pellets once daily (200 g/day; sheep pellets based on wheat, millrun and lucerne; 17.5% protein, 2.5% fat, 17% fibre, 20% ADF, 34% NDF) and lucerne hay twice daily. Ewes in the main trial were supplemented with thiamine powder mixed in with their daily feed when thiamine deficiency symptoms appeared in this cohort (see results Section 3.3. Main Trial).

Ewes were left undisturbed while lambing, unless there were obvious signs of distress, foetal malpresentation (one leg back, two legs back, breech), or there was no progress achieved 1 h after foetal membranes or foetal parts appeared. If assistance was given, the ewe was restrained in a lateral lying position by one handler, and progression of the lamb through the birth canal was assisted by another handler. The ewe and lamb were monitored closely until the ewe was observed to be grooming the lamb and allowing it to suckle.

Once a ewe had lambed in the lambing enclosure, time of birth and birth intensity (eutocic/normal or dystocic/difficult) were documented from the video recordings. Birth intensity was determined by a single observer familiar with lambing ewes. Ewe and lamb pairs that appeared fit, healthy and well bonded were moved into an adjacent paddock, or alternatively to hospital pens to allow treatment of ewes or to facilitate bonding. The ewe was replaced by a pregnant ewe from the holding paddock, keeping the number of ewes in the lambing enclosure at 20. This was continued until all ewes had lambed.

### 2.2. Blood Sampling 

Blood samples were collected by jugular venepuncture into 10 mL lithium heparin vaccutainer tubes. Samples were analysed with the iStat-1 handheld analyser (Abbot Point of Care, Princeton, NJ, USA) using a combination of cartridges (Table 1). Cartridge types were changed for time course and main trials due to changes in availability. 

Peripheral blood glucose was measured in the Time Course and Main Trial phases, using an AccuCheck handheld glucometer (AccuChek, Roche Diabetes Care, North Ryde, NSW, Australia) via capillary puncture in the ear.

### 2.3. Pilot Trial

The pilot trial was conducted over three lambing cohorts in 2018. A total of 37 ewes were sampled for the pilot trial from a total of 420 lambing ewes. The first cohort was lambed in April–May (blood samples collected from 15 out of 221 ewes; *n* = 15; 13 single/2 twin births), the second in August–September (*n* = 100; blood samples collected from 10 out of 100 ewes; *n* = 10; 9 single/1 twin births), and the third in October–November (blood samples collected from 12 out of 99 ewes; *n* = 12; 7 single/5 twin births). For the first cohort, if a ewe was found less than 2 h post-lambing, she was eligible for a blood sample. The ewe and her lamb(s) were brought into the lambing shed, a blood sample was collected, and the ewe and her lamb(s) were then moved to the adjacent paddock.

For the subsequent two cohorts, timing of blood sampling was adjusted to allow sufficient time to bond for ewe and lamb. Hence, blood samples were collected 2–4 h post-birth and a second sample was added at 24 h post-birth. Analysis of the samples was as described above. 

### 2.4. Time Course

Twelve ewes of a lambing cohort of 100 ewes were selected for a blood sampling time course. All ewes were carrying singleton pure Merino lambs. At two time points during gestation—mid gestation approximately 80 days gestation and late gestation approximately 140 days gestation—all ewes of the cohort (*n* = 100) were brought into the yards for blood glucose determination. Additionally, the ewes selected for indoor lambing had a blood sample collected for analysis with the iStat analyzer (for cartridge details, see Table 1). 

The six ewes first due to lamb were brought into an indoor group lambing pen (4 m × 3 m) with straw bedding and two day/night surveillance cameras. Ewes were provided with free access to water, ad libitum lucerne hay and a daily ration of sheep pellets and fava beans, as above.

The ewes were checked every 2–3 h for signs of lambing, and when the first signs appeared, the ewe was monitored every 30 min until birth. Once a ewe had lambed, she was moved with her lamb into a single pen (1.5 m × 1.5 m) adjacent to the lambing pen. A blood sample was collected within 2 h of lambing and blood glucose was recorded. This protocol was repeated at 4 h, 8 h, 24 h and 48 h post-lambing. For ewes that lambed outside the observation period, no further blood samples were collected. After all blood was collected, the ewe and lamb were moved into a paddock with other ewes and lambs, and one of the other selected pregnant ewes was brought into the indoor lambing pen.

Only complete sample sets for all time points were used for the time course analysis. A complete sample set was obtained from a total of six ewes for the time course of iStat analysis. 

### 2.5. Main Trial 

At two time points during gestation, at 80 days post-joining for mid-gestation, and at 140 days post-joining for late-gestation, ewes (*n* = 100) were brought into the yards for blood glucose determination. One week before lambing was due to commence, the 20 ewes that were expected to lamb first based on foetal size at pregnancy scanning were brought into the lambing shed, had their blood glucose measured and recorded and a blood sample was collected. The rest of the ewes were brought into the lambing shed in groups of 20 (based on due date) in two- or five-day intervals for the same blood sampling protocol. This method was employed to reduce stress on the ewes and to allow sampling of all ewes at around one week before lambing.

Some ewes in this trial showed symptoms of thiamine deficiency in the first week of observation. The likely cause was nutritional management of the ewes before entering the trial, which was affected by severe drought conditions in 2018/2019. Under these conditions, diets were low in fibre and high in pulses and grains. Over prolonged periods of time, this can change the rumen biome affecting thiamine production. No symptoms had been diagnosed up until the end of pregnancy and it is hypothesised that the additional thiamine demands of the growing foetus may have triggered the symptoms in previously borderline deficient ewes. Once diagnosed by a veterinarian, all ewes were supplemented with thiamine powder with hard feed every day. All ewes were given a thiamine intramuscular injection, and ewes that were acutely symptomatic were given thiamine intravenously. Treated ewes became ineligible for further sample collection. Overall, 11 out of 100 ewes were treated for acute thiamine deficiency. Of the 11 treated ewes, 6 ewes recovered and 5 ewes were euthanised by captive bolt stun and exsanguination as per the approved protocol. Any ewe that was found within 3 h of lambing was eligible for a birth blood sample. She was brought into the lambing shed with her lamb, a blood sample was collected and blood glucose was recorded. She was then either penned outside or returned to the lambing enclosure, depending on evident secure ewe–lamb bonding and appropriate maternal behaviour. On the following day, between 16 h and 26 h post-lambing, a second sample was collected from the ewe. The ewe and lamb were then moved to an adjacent paddock.

If a ewe with a newborn lamb had not been found until more than 3 h after lambing, most commonly on the following morning, a single post-birth blood sample was collected at this time, at 16 h to 26 h after lambing and then ewe and lamb were released into the adjacent paddock.

Blood samples were successfully collected at birth, within 3 h of birth, from 27 ewes (*n* = 27; 17 single/10 twin births) and on the day following birth within 16–26 h from 30 ewes (*n* = 30; 18 single/12 twin births).

### 2.6. Statistical Analysis

All data were recorded on paper and then entered into MS Excel at the end of the trial (Microsoft, Redmont, WA, USA). Excel was used to clean the data, organise it and perform basic descriptive statistics (mean, range, standard deviation).

Statistical tests were performed using Genstat (VSN International, Hemel Hempstead, UK, 2021). Unpaired Student’s t-Tests were used to compare blood marker values between eutocic and dystocic births, and for comparing mid-gestation values and subsequent values in the time course. The significance level was *p* < 0.05.

## 3. Results

All ewes in this study, including dystocic ewes, successfully delivered their lambs unassisted within the time limits allowed by the study protocol. 

### 3.1. Pilot Trial

In the first lambing cohort, 15 ewes were sampled 0–2 h after birth from eutocic (*n* = 11; 9 single/2 twin births) and dystocic (*n* = 4; 4 single/0 twin births) lambing events. This cohort was analysed separately to cohorts 2 and 3 due to changes made to the sampling protocol. Potassium (eutocic = 4.07 ± 0.33 mmol/L, dystocic = 3.85 ± 0.13 mmol/L; *p* = 0.04) and chloride (eutocic = 114.27 ± 2.24 mmol/L, dystocic = 109.25 ± 3.30 mmol/L; *p* = 0.02) were significantly lower in dystocic ewes. Additionally, TCO_2_ (eutocic = 23.64 ± 1.21 mmol/L, dystocic = 27.75 ± 2.99 mmol/L; *p* = 0.03), creatinine (eutocic = 0.76 ± 0.10 mg/dL, dystocic = 1.00 ± 0.082 mg/dL; *p* = 0.001), base excess (eutocic = -0.18 ± 1.33 mmol/L, dystocic = 5.75 ± 4.86 mmol/L; *p* = 0.05) and HCO_3_ (eutocic = 23.20 ± 1.60 mmol/L, dystocic = 28.90 ± 4.80 mmol/L; *p* = 0.05) were all significantly higher in dystocic ewes.

In the second cohort, 10 ewes were sampled 2–4 h after birth, and again at 24 h after birth from eutocic (*n* = 7; 6 single/1 twin births) and dystocic (*n* = 3; 3 single/0 twin births) lambing events. In the third cohort, 12 ewes were sampled 2–4 h after birth, and again at 24 h after birth from eutocic (*n* = 10; 6 single/4 twin births) and dystocic (*n* = 2; 1 single/1 twin births) lambing events. When data from cohorts 2 and 3 were analysed together (17 eutocic ewes and 5 dystocic ewes), no differences were seen between eutocic and dystocic ewes at the first time point 2–4 h after birth for any blood parameters measured. At the second time point 24 h after birth, sO_2_ (eutocic = 83.65 ± 8.04, dystocic = 89.90 ± 3.42; *p* = 0.01) was significantly higher in dystocic ewes. 

Cohorts 2 and 3 lambed in late winter/early spring and late spring, respectively, of the Australian New England climate. To address the question of whether environmental conditions had an effect on the obtained results, data obtained for cohorts 2 and 3 were also analysed separately. When analysed separately, at the first time point for cohort 2, creatinine (eutocic = 0.64 ± 0.098 mg/dL, dystocic = 0.47 ± 0.058 mg/dL; *p* = 0.005) was significantly lower in dystocic ewes; however, glucose (eutocic = 129.57 ± 28.57 mg/dL, dystocic = 90.33 ± 35.73 mg/dL; *p* = 0.09) and potassium (eutocic = 4.37 ± 0.37 mmol/L, dystocic = 3.70 ± 0.56 mmol/L; *p* = 0.08) were not significantly different. At 24 h after birth, TCO_2_ (eutocic = 25.00 ± 2.00 mmol/L, dystocic = 28.00 ± 2.00 mmol/L; *p* = 0.05) was significantly higher in dystocic ewes. 

At the first time point of cohort 3, calcium (eutocic = 1.16 ± 0.12 mmol/L, dystocic = 1.29 ± 0.042 mmol/L; *p* = 0.02) was significantly higher in dystocic ewes. At 24 h after birth, chloride (eutocic = 109.70 ± 3.23 mmol/L, dystocic = 112.50 ± 0.71 mmol/L; *p* = 0.02) and sO_2_ (eutocic = 79.2 ± 5.63%, dystocic = 87.00 ± 2.83%; *p* = 0.03) were significantly higher, and calcium (eutocic = 1.16 ± 0.18 mmol/L, dystocic = 0.95 ± 0.028 mmol/L; *p* = 0.003), TCO_2_ (eutocic = 24.50 ± 2.27 mmol/L, dystocic = 22.50 ± 0.71 mmol/L; *p* = 0.03), pCO_2_ (eutocic = 33.62 ± 3.59 mmHg, dystocic = 28.95 ± 1.06 mmHg; *p* = 0.006), base excess (eutocic = 1.40 ± 3.44 mmol/L, dystocic = −1.00 ± 0.00 mmol/L; *p* = 0.03) and HCO_3_ (eutocic = 24.83 ± 3.06 mmol/L, dystocic = 22.40 ± 0.42 mmol/L; *p* = 0.02) were significantly lower in dystocic ewes. 

Within each cohort, there were parameters differentiating between eutocic and dystocic ewes. There was no unifying trend across these cohorts due to the small sample sizes. 

### 3.2. Time Course

During pregnancy, all 100 ewes of the larger cohort, to which the ewes involved in the time course experiment belonged, had blood glucose measured twice by the test strip method: once at 80 days gestation and again at 140 days gestation (mid-and late gestation, respectively). Glucose was significantly higher at mid-gestation compared to late gestation (mid = 4.20 ± 0.75, late = 3.45 ± 1.05; *p* < 0.001). There was no difference found between dystocic and eutocic ewes. 

**Figure 1:**
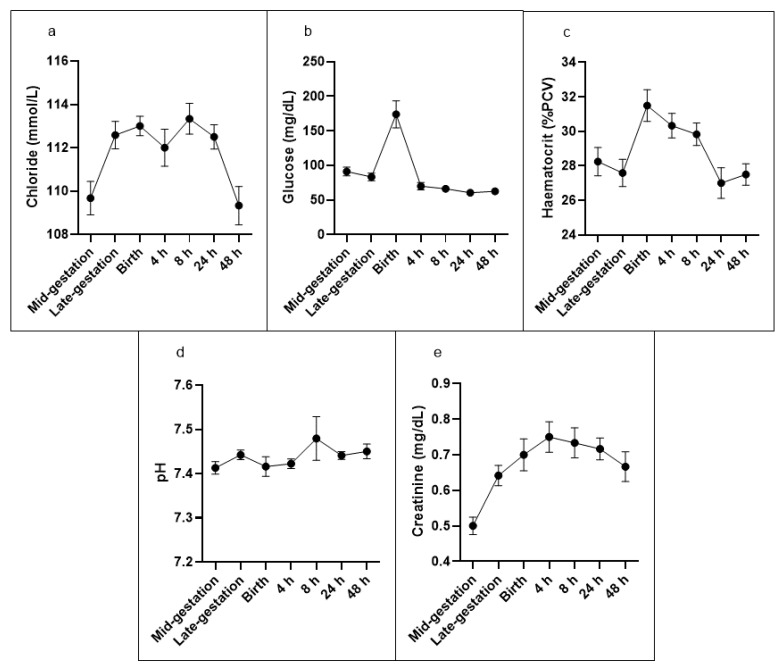
Average values (s.d. error bars) of 6 single bearing ewes at two time points before birth and five time points after birth for selected blood markers; (**a**) Average chloride (mmol/L), (**b**) Average glucose (mg/dL), (**c**) Average haematocrit (%PCV), (**d**) Average pH, (**e**) Average creatinine (mg/dL).

All other results reported here were derived from iStat cartridge analysis (Table 2). Complete datasets were obtained from 6 out of the selected 12 ewes for the time course experiment. The anion gap decreased at late gestation, returned to mid-gestation at birth, decreased again from 4 h and remained lower than at mid-gestation up to 48 h. Potassium increased by 8 h after birth but returned to mid-pregnancy levels thereafter. Urea decreased at birth, remained lower than mid-pregnancy at 4 h after birth and then returned to mid-pregnancy level. Chloride increased at late gestation and birth, returned to mid-pregnancy level at 4 h, but increased again at 8 h and 24 h (Figure 1a). Glucose was higher at birth and nearly doubled compared to the mid-gestation level. After birth, glucose dropped and remained significantly lower than mid-gestation for the remainder of the observation period (Figure 1b). 

Haemoglobin and haematocrit (Figure 1c) were higher by 11% at birth but were not different at any other time point. pH (Figure 1d) and pCO_2_ remained unchanged throughout the entire observation period.

Creatinine concentration was higher at every time point compared to mid-gestation. It reached a peak at 4 h post-birth and declined thereafter but remained elevated for the entire observation period (Figure 1e). Sodium, TCO_2_, pCO_2_, HCO_3_, base excess, pH (Figure 1d) and pCO_2_ remained unchanged throughout the entire observation period (Table 2).

### 3.3. Main Trial 

For this trial, samples were collected from all ewes (*n* = 100) at 80 d and 140 d gestation for peripheral blood glucose determination. No differences were observed between singleton or twin-bearing ewes and ewes that would later experience eutocic or dystocic birth. 

The full set of blood parameters was measured approximately one week before parturition for all ewes, except for seven individuals (*n* = 93; including 84 eutocic (54 single/30 twin births) and 9 dystocic (6 single/3 twin births) events); five ewes were affected by ‘stargazing’ disease/thiamine deficiency (see above; one from each group), one ewe died before sampling (Group 4) and one ewe was excluded as she was the only ewe delivering triplets (Group 2). At birth, blood samples were collected within 3 h of birth (*n* = 27), including 19 eutocic (11 single/8 twin births) and 8 dystocic (6 single/2 twin births) events. Samples from the day following birth were collected within 16–26 h from a total of 30 ewes, including 21 eutocic events (21 single/13 twin births) with samples collected at 16 h 25 min–25 h 42 min postpartum (1224 ± 163 minutes) and 9 dystocic events (6 single/3 twin births) with samples collected at 16 h 10 min–26 h 04 min postpartum (1253 ± 231 minutes). 

Twin-bearing ewes were represented in 35% (33/93) of pre-birth samples (30/84 or 36% of eutocic, and 3/9 or 33% of dystocic samples); 37% (10/27) of samples collected at birth (8/19 or 42% of eutocic and 2/8 or 25% dystocic ewes); and 37% (16/43) of samples collected at 16–26 h post birth (13/34 or 38% of eutocic and 3/9 or 33% of dystocic samples). Hence, twin-and singleton-bearing ewes were represented consistent with the overall average in pre-birth and post-birth samples. At birth, twin-bearing ewes were slightly underrepresented in the total sample set as one dystocic twin-bearing ewe lambed outside of the daily observation period. Overall, twin-bearing ewes were not overrepresented in the samples, and therefore, in the analysis of blood sample data, birth type (singleton or twin) was not further considered as a factor. 

Before lambing, dystocic ewes had a significantly lower chloride concentration compared to eutocic ewes (eutocic = 113.84 ± 2.43 mmol/L, dystocic = 111.63 ± 1.77 mmol/L; *p* = 0.014). Additionally, dystocic ewes had a higher haematocrit (eutocic = 27.42 ± 2.69% PCV, dystocic = 29.88 ± 3.87%PCV; *p* = 0.035) and a higher haemoglobin concentration (eutocic = 9.32 ± 0.91 g/dL, dystocic = 10.16 ± 1.31 g/dL; *p* = 0.033) compared to eutocic ewes.

Blood glucose concentration was higher at birth for eutocic ewes compared to dystocic ewes, but the difference was not significant (eutocic = 165.37 ± 44.17 mg/dL, dystocic = 127.75 ± 54.74 mg/dL; *p* = 0.07). Blood creatinine concentration was significantly higher at birth for dystocic ewes than eutocic ewes (eutocic = 0.79 ± 0.14 mmol/L, dystocic = 0.93 ± 0.18 mmol/L; *p* = 0.02).

In the sampling period 16–26 h after birth, glucose and creatinine concentrations were not significantly different between eutocic and dystocic ewes; the values were comparable to pre-birth concentrations. Dystocic ewes had a lower pH (7.41 +/− 0.089) than eutocic ewes (91 7.47 +/− 0.079), with a corresponding difference in base excess between eutocic (−1.38 +/− 2.80 mmol/L) and dystocic ewes (−3.44 +/− 2.55 mmol/L).

In summary, before birth, dystocic ewes presented with significantly lower chloride and higher haematocrit and haemoglobin compared to eutocic ewes. Within 3 h of birth, blood glucose was significantly lower, and creatinine was significantly higher in dystocic ewes. In the 16–26 h after birth, pH and base excess parameters were significantly lower in dystocic ewes.

## 4. Discussion

During the pilot trial, we aimed to establish whether we could observe indicators promising to distinguish between dystocic or eutocic lambing events in blood samples collected from ewes in the peri-natal time period and analysed with pen-side diagnostics. 

In the first cohort, we found that potassium and chloride were lower in dystocic ewes. It is well documented that potassium and chloride are key ions involved in myocyte excitation and uterine contractility, allowing the proper progression of labour [13]. Disturbances in the concentrations and activity of these ions may lead to adverse labour events, including dystocia [14]. The disturbances we found in chloride and potassium may have existed before birth and influenced birth difficulty, or they may have been an effect of dystocia itself. Without pre-birth sampling, as suggested by [15] this question could not be addressed in the pilot trial.

Maternal acid–base function and dysfunction are well documented in humans (see reviews by [16,17]. Several studies, including [18,19], have shown that prolonged or dystocic labours have a greater risk of metabolic disturbance, notably decreased blood pH and oxygen saturation, as well as increased lactate (i.e., acidosis). In cohort 1, we found that dystocic ewes presented with higher TCO_2_, base excess and HCO_3_ levels than eutocic ewes. This could be a compensatory effect, counteracting the acidosis that may have occurred during labour. Additionally, creatinine was higher in dystocic ewes. This was expected, as creatinine is a by-product of muscle and protein metabolism [20]. Elevations in blood creatinine concentration were described in dystocic camels [15,21]; however, the results were not significant.

When analysed separately, cohorts 2 and 3 returned conflicting results. This may be due to seasonal differences in nutrition, as cohort 2 lambed in late winter and cohort 3 in mid-spring, or the small sample size in each cohort. When the data of cohorts 2 and 3 were combined and analysed together, there were no significant differences between eutocic and dystocic lambing events for any blood parameters analysed for samples taken 2–4 h after birth. The adaptive stress response, especially for acute stressors—including birth—is a fast-acting system that keeps the body systems in eustasis [22], and it is possible that the changes in blood parameters seen at birth in the first cohort were resolved by the time samples were collected in the subsequent two cohorts with its slightly modified sampling protocol. In sheep, Comline and Silver [23] described changes in maternal glucose, lactic acid and free fatty acids occurring at birth, but quickly resolving in the following 2–4 h.

In summary, we concluded from the pilot trial that it may be possible to identify pen-side indicators of dystocia within experimental cohorts. 

To refine our sampling protocol, a time course analysis was next undertaken. We aimed to establish a suitable time window for the first blood sample collected after birth, considering the need to allow the establishment of ewe–lamb bonds and anticipating feasible collection protocols for the main trial. We also aimed to establish the potential value of a blood sample collected within 48 h, which can be realistic in field conditions. Blood samples were collected from 12 Merino ewes carrying single Merino lambs at two time points before birth, at approximately 80 days and 140 days of gestation, and of six of those ewes that had a normal birth, within 2 h of birth and then at 4 h, 8 h, 24 h and 48 h after birth.

A pronounced elevation in glucose was seen in all ewes at birth, followed by a rapid decrease back to pre-birth concentration by the time the 4 h sample was collected. A fundamental study by Comline and Silver [23] described the changes in blood markers of ewes over time in late pregnancy, at parturition, and in the hours following birth. Similarly, they described glucose as the most obvious change, with very pronounced hyperglycaemia occurring at birth. Additionally, they reported little change in pCO_2_ and pH, which was also observed in the results presented here.

Creatinine is a by-product of protein metabolism and can increase as a result of muscle work or damage [20], thus it is expected to rise after an intense period of work, such as parturition. In the time course results, we saw a gradual increase in blood creatinine from mid-gestation through birth and a peak at 4 h after birth. Creatinine then declined slowly to the end of the sampling protocol. These results mimic those reported during pregnancy and lactation of ewes [24] and during the early postpartum period of bitches [25]. It should be noted that all time points fell within the normal range of blood creatinine concentration for sheep [26]; periparturient creatinine concentrations changed but were not abnormal.

An increase at birth was also observed in haematocrit, with PCV slowly returning to pre-birth levels, which mirrors the trend described in bitches [25].

From the time course and from our pilot trial, we determined that a pre-birth sample was necessary to capture the blood profile before lambing, and any possible indicators of birth difficulty could be recognised. A sample collected within 2–3 h of birth would capture the disturbance caused by birth, and any irregularities caused by a difficult birth should be present and identifiable. Finally, a sample should be taken 16–26 h after birth, as some important markers such as creatinine take longer to normalise, and differences may still be observable as a result of dystocia. This relatively wide time range was chosen to avoid exclusion of sample sets for which a narrower time range would have precluded collection of the second time point for logistical reasons.

In the main trial, pre-birth blood samples were collected from all ewes around one week before lambing to serve as a pre-birth baseline. We also collected as many blood samples as possible within 3 h of birth and again 16–26 h after birth.

At 140 days of pregnancy, dystocic ewes had significantly lower chloride than eutocic ewes. This result indicates that pre-birth disturbance or insufficiency of chloride may have an effect on birth difficulty. As chloride is essential for myometrial excitation, contractility and labour progression, a decrease in available chloride may decrease the strength of contractions and increase the risk of adverse labour events [13,27,28].

Dystocic ewes had significantly higher haematocrit and haemoglobin levels. Increased blood volume and a smaller increase in PCV (haemodilution) during gestation is to be expected; as maternal body weight increases, plasma volume increases [29]. This phenomenon has been seen in many species and was described in goats by [30]. Higher PCV in dystocic cows just before surgical intervention was seen by [31]. They also described a more pronounced increase in haemoglobin in dystocic cows. They hypothesised dehydration and excitation (releasing catecholamines) were the driving mechanisms behind these differences.

At birth, blood glucose concentration was lower in dystocic ewes, but the difference was not significant. Larger group sizes and an earlier collection time will be required to address whether the observed difference has significance. Based on our time course results, the chosen at-birth collection time point may have missed the period of perinatal elevation, which may show differences between dystocic and eutocic ewes and epistatic mechanisms that tightly control blood glucose levels may have adjusted this difference already, if it had been there. Glucose appears to be the main energy source for myometrial cells [32,33]. During birth, glucose is mobilised via increased cortisol and adrenaline as a result of pain and stress [34], ensuring the myometrium has enough energy to fuel the intense activity of birth. Generally, the peak in glucose is at birth, or soon thereafter [23,35]. Interestingly, many studies have reported an increase in glucose in dams that have experienced dystocia [15,25,34,36]; however, many, if not all, had surgical or manual intervention after a certain threshold, whereas the ewes in this study were able to successfully lamb unassisted within the time boundaries set for the study.

Creatinine concentration was significantly higher in dystocic ewes compared with eutocic ewes. Creatinine levels rise during and after exercise as a result of exercise-induced muscle breakdown [37], and parturition is an event characterised by intense myometrial activity, so it is reasonable to assume that creatinine would be elevated after birth. Prolonged or dystocic births are extended periods of myometrial activity and therefore creatinine concentrations would be elevated further. Across several studies and species, such as the camel [15,21], buffalo [36] and dog [25], creatinine appears trend higher in dystocic dams; however, their results were not statistically significant. Again, in this study, dystocic ewes were ultimately able to successfully lamb unassisted, whereas in other studies intervention may have precluded elevation of creatinine. 

By the time blood samples were collected at 16–26 h post-birth, most blood parameters had normalised, so there were no significant differences between eutocic and dystocic ewes. This further supports the idea expressed earlier that the acute adaptive stress response quickly regulates the body back to eustasis [22]. An exception to this were pH and base excess, which were lower for dystocic ewes. This result matched the result found earlier in our first pilot trial cohort. As discussed above, acid–base balance may be affected by lactic acidosis as a result of anaerobic energy creation in the muscle during the physical exertion of prolonged labour. More work will be required to confirm whether this parameter can be used reliably to identify dystocic ewes.

For creatinine, which remains overall elevated for an extended time period after birth, we could not establish differences between eutocic and dystocic ewes. A more narrowly defined time window for collection of the second post-birth sample may have allowed such differentiation and more work will be required to address this possibility.

In this study, we restricted the investigated parameters to those readily assessable through pen-side diagnostics. To get a more complete understanding of differences in the physiology of dystocic and eutocic ewes, analysis of additional metabolic markers, such as beta-hydroxy-butyrate and non-esterised fatty acids, may be of interest [38]. Further studies will be needed to confirm the results presented here in repeat large-scale studies. 

## 5. Conclusions

In this series of trials, we aimed to use pen-side analysis techniques to collect information on blood parameters in pregnant and periparturient Merino ewes to further understand the effect of dystocia on the physiology of the ewe. Pilot study results indicated that observable differences can be found between dystocic and eutocic ewes. The observed differences in the pilot study were dependent on cohorts, pointing to the strong influence of environmental conditions on individual parameters. 

The main trial confirmed observable differences between normal and difficult lambing events in the pre- and postpartum periods. Dystocic ewes presented in late pregnancy with significantly lower chloride and higher haematocrit and haemoglobin, compared to eutocic ewes. At birth, creatinine was significantly higher in dystocic ewes. On the day following birth, acid–base balance was affected in dystocic ewes. The observed differences may have potential as diagnostic markers. 

This study has demonstrated the potential benefits of pen-side diagnostics to predict and identify dystocic ewes. More research will be needed to confirm these results and to further our understanding of the underlying factors contributing to incidences of dystocia in sheep production systems.

## Figures and Tables

**Table 1:** Blood analysis parameters by trial.

Cartridge Type	Blood Parameters Analysed	Trial Used
CG4+	Na, K, Cl, Ca (as ionised calcium), TCO_2_, Glucose, Urea, Creatinine, Haematocrit (PCV), Haemoglobin, Anion Gap	Pilot
CHEM8+	pH, pCO_2_, pO_2_, Base Excess, HCO_3_, TCO_2_, sO_2_, Lactate	Pilot
EC8+	Na, K, Cl, TCO_2_, Urea, Glucose, Haematocrit (PCV), pH, pCO_2_, HCO_3_, Base Excess, Anion Gap, Haemoglobin	Time Course, Main Trial
Crea	Creatinine	Time Course, Main Trial

**Table 2:** Average (s.d.) values for investigated blood parameters measured at mid-gestation, late gestation, birth and 4 h, 8 h, 24 h, and 48 h post-birth. Comparisons are between mid-gestation and subsequent time points.

Blood Parameter	Mid-Gestation	Late-Gestation	Birth	4 h	8 h	24 h	48 h
Sodium (mmol/L)	149.67 (1.67)	150.00 (1.21)	150.17 (1.72)	149.83 (2.40)	148.83 (1.33)	149.50 (2.43)	149.67 (2.07)
Potassium (mmol/L)	4.04 (0.40)	3.66 (0.28)	4.15 (0.37)	4.30 (0.45)	4.77 (0.36) **	4.40 (0.22)	4.23 (0.39)
Chloride (mmol/L)	109.67 (2.67)	112.58 (2.19) *	113.00 (1.10) **	112.00 (2.10)	113.33 (1.75) **	112.50 (1.38) *	112.19 (2.16)
Anion Gap (mmol/L)	20.33 (1.61)	17.17 (1.99) **	19.83 (2.71)	17.83 (1.33) **	17.00 (1.55) ***	16.83 (1.60) ***	18.17 (0.84) **
Glucose (mg/dL)	91.17 (21.87)	83.33 (19.54)	173.83 (47.94) ***	70.00 (13.01) *	66.17 (3.43) **	60.33 (5.57) **	62.50 (10.09) **
Urea (BUN) (mmol/L)	28.42 (4.68)	29.58 (3.73)	19.83 (3.60) ***	22.50 (4.32) **	25.50 (3.51)	25.33 (2.94)	25.19 (4.23)
Haematocrit (% PCV)	28.25 (2.86)	27.58 (2.75)	31.50 (2.26) *	30.33 (1.75)	29.83 (1.60)	27.00 (2.19)	29.09 (1.52)
Haemoglobin (g/dL)	9.61 (0.98)	9.33 (0.97)	10.72 (0.76) *	10.30 (0.58)	10.13 (0.55)	9.18 (0.73)	9.87 (0.53)
pH (standard unit)	7.41 (0.05)	7.44 (0.04)	7.42 (0.05)	7.42 (0.03)	7.48 (0.12)	7.44 (0.02)	7.44 (0.04)
pCO_2_ (mmHg)	37.59 (4.81)	34.87 (2.53)	34.05 (8.21)	37.32 (3.75)	34.82 (3.06)	36.28 (2.65)	35.83 (3.89)
TCO2 (mmol/L)	25.00 (2.22)	24.92 (2.19)	22.67 (4.80)	25.50 (2.26)	24.17 (1.83)	25.67 (1.21)	24.65 (1.17)
HCO3 (mmol/L)	23.88 (2.08)	23.83 (2.27)	21.70 (4.34)	24.33 (2.11)	22.97 (1.82)	24.67 (0.96)	23.56 (1.28)
Base Excess (mmol/L)	−0.83 (2.41)	−0.25 (2.67)	−2.83 (4.26)	0.00 (2.45)	−1.33 (1.75)	0.67 (0.82)	−0.76 (1.72)
Creatinine (mg/dL)	0.50 (0.085)	0.64 (0.097) **	0.70 (0.11) ***	0.75 (0.10) ***	0.73 (0.10) ***	0.72 (0.075) ***	0.67 (0.10) **

* *p* < 0.05, ** *p* < 0.01, *** *p* < 0.001.

## Data Availability

Data collected in this study have not been publicly archived yet. Data can be accessed by contacting the corresponding author.

## References

[1] Refshauge G., Brien F.D., Hinch G.N., Van De Ven R. (2016). Neonatal lamb mortality: Factors associated with the death of Australian lambs. Anim. Prod. Sci..

[2] Jacobson C., Bruce M., Kenyon P.R., Lockwood A., Miller D., Refshauge G., Masters D.G. (2020). A review of dystocia in sheep. Small Rumin. Res..

[3] Hinch G.N., Brien F. (2014). Lamb survival in Australian flocks: A review. Anim. Prod. Sci..

[4] Kenyon P.R., Pain S.J., Hutton P.G., Jenkinson C.M.C., Morris S.T., Peterson S.W., Blair H.T. (2011). Effects of twin-bearing ewe nutritional treatments on ewe and lamb performance to weaning. Anim. Prod. Sci..

[5] Mulvaney F.J., Kenyon P.R., Morris S.T., West D.M. (2008). Ewe lamb nutrition during pregnancy affects pregnancy outcome. Aust. J. Exp. Agric..

[6] Bunter K.L., Swan A.A., Gurman P.M., Brown D.J. (2021). New genomically enhanced reproduction breeding values for Merino sheep allow targeted selection for conception rate, litter size and ewe rearing ability. Anim. Prod. Sci..

[7] Brien F.D., Hebart M.L., Smith D.H., Edwards J.E.H., Greeff J.C., Hart K.W., Refshauge G., Bird-Gardiner T.L., Gaunt G., Behrendt R. (2010). Opportunities for genetic improvement of lamb survival. Anim. Prod. Sci..

[8] Ahmed M.H., Wilkens M.R., Möller B., Ganter M., Breves G., Schuberth H.-J. (2020). Blood leukocyte composition and function in periparturient ewes kept on different dietary magnesium supply. BMC Veter. Res..

[9] Morris J., Fernandez J., Chapa A., Gentry L., Thorn K., Weick T. (2002). Effects of sample handling, processing, storage, and hemolysis on measurements of key energy metabolites in ovine blood. Small Rumin. Res..

[10] Chong S.K., Reineke E.L. (2016). Point-of-Care Glucose and Ketone Monitoring. Top. Companion Anim. Med..

[11] Fletcher D.J. (2016). Point of Care Testing in Small Animal Practice: Opportunities and Challenges. Top. Companion Anim. Med..

[12] Smith D., McNally J., Little B., Ingham A., Schmoelzl S. (2020). Automatic detection of parturition in pregnant ewes using a three-axis accelerometer. Comput. Electron. Agric..

[13] Dunford J.R., Blanks A.M., Gallos G. (2019). Calcium activated chloride channels and their role in the myometrium. Curr. Opin. Physiol..

[14] Brainard A.M., Korovkina V.P., England S.K. (2007). Potassium channels and uterine function. Semin. Cell Dev. Biol..

[15] Ghoneim I., Waheed M., Al-Eknah M., Al-Raja’A A. (2016). Effect of dystocia on some hormonal and biochemical parameters in the one-humped camel (*Camelus dromedarius*). Theriogenology.

[16] Arrowsmith S., Kendrick A., Hanley J.-A., Noble K., Wray S. (2014). Myometrial physiology—Time to translate?. Exp. Physiol..

[17] Omo-Aghoja L. (2014). Maternal and fetal acid-base chemistry: A major determinant of perinatal outcome. Ann. Med. Health Sci. Res..

[18] Musaba M.W., Barageine J.K., Ndeezi G., Wandabwa J.N., Weeks A. (2019). Effect of preoperative bicarbonate infusion on maternal and perinatal outcomes of obstructed labour in Mbale Regional Referral Hospital: A study protocol for a randomised controlled trial. BMJ Open.

[19] Quenby S., Pierce S.J., Brigham S., Wray S. (2004). Dysfunctional Labor and Myometrial Lactic Acidosis. Obstet. Gynecol..

[20] Salazar M.J.H. (2014). Overview of Urea and Creatinine. Lab. Med..

[21] Ali A., Derar D., Tharwat M., Zeitoun M.M., Al-Sobyil F.A. (2016). Dystocia in dromedary camels: Prevalence, forms, risks and hematobiochemical changes. Anim. Reprod. Sci..

[22] Dhabhar F.S. (2018). The short-term stress response—Mother nature’s mechanism for enhancing protection and performance under conditions of threat, challenge, and opportunity. Front Neuroendocrinol..

[23] Comline R.S., Silver M. (1972). The composition of foetal and maternal blood during parturition in the ewe. J. Physiol..

[24] El-Sherif M., Assad F. (2001). Changes in some blood constituents of Barki ewes during pregnancy and lactation under semi arid conditions. Small Rumin. Res..

[25] Simões C.R.B., Vassalo F.G., Lourenço M.L.G., de Souza F.F., Oba E., Sudano M.J., Prestes N.C. (2016). Hormonal, Electrolytic, and Electrocardiographic Evaluations in Bitches With Eutocia and Dystocia. Top. Companion Anim. Med..

[26] Desco M., Cano M.J., Duarte J., Rodriguez F., Fernández-Caleya D., Alvarez-Valdivielso M., Antoranz J.C., Rubio M.A., García-Barreno P., del Cañizo J.F. (1989). Blood biochemistry values of sheep (Ovis aries ligeriensis). Comp. Biochem. Physiol. A Comp. Physiol..

[27] Bernstein K., Vink J.Y., Fu X.W., Wakita H., Danielsson J., Wapner R., Gallos G. (2014). Calcium-activated chloride channels anoctamin 1 and 2 promote murine uterine smooth muscle contractility. Am. J. Obstet. Gynecol..

[28] Jones K., Shmygol A., Kupittayanant S., Wray S. (2004). Electrophysiological characterization and functional importance of calcium-activated chloride channel in rat uterine myocytes. Pflugers Arch..

[29] O’Sullivan J.F. (1960). Anaemia in Pregnancy. BMJ.

[30] Iriadam M. (2007). Variation in certain hematological and biochemical parameters during the peri-partum period in Kilis does. Small Rumin. Res..

[31] Tiwari P., Gupta H.P., Prasad S., Sheetal S.K. (2020). Effect of Different Surgical Approaches in Dystocia on Different Hematological Parameters Before and After Caesarean Section in Cows. Vet. Res..

[32] Steingrímsdóttir T., Ronquist G., Ulmsten U. (1993). Energy economy in the pregnant human uterus at term: Studies on arteriovenous differences in metabolites of carbohydrate, fat and nucleotides. Eur. J. Obstet. Gynecol. Reprod. Biol..

[33] Rizzo A., Angioni S., Spedicato M., Minoia G., Mutinati M., Trisolini C., Sciorsci R.L. (2010). Uterine contractility is strongly influenced by steroids and glucose metabolism: Anin vitrostudy on bovine myometrium. Gynecol. Endocrinol..

[34] Vannucchi C.I., Rodrigues J.A., Silva L.C.G., Lúcio C.F., Veiga G.A.L., Furtado P.V., Oliveira C.A., Nichi M. (2015). Association between birth conditions and glucose and cortisol profiles of periparturient dairy cows and neonatal calves. Vet. Rec..

[35] Chew C.S., Rinard G.A. (1979). Glycogen Levels in the Rat Myometrium at the End of Pregnancy and Immediately Postpartum. Biol. Reprod..

[36] Amin Y.A., Noseer E.A., Abu El-Naga E.M. (2020). Changes in the Fetal Fluids Compositions during Dystocia of Dairy Buffaloes. Adv. Anim. Vet. Sci..

[37] Bongers C.C.W.G., Alsady M., Nijenhuis T., Tulp A.D.M., Eijsvogels T.M.H., Deen P.M.T., Hopman M.T.E. (2018). Impact of acute versus prolonged exercise and dehydration on kidney function and injury. Physiol. Rep..

[38] González-García E., Tesniere A., Camous S., Bocquier F., Barillet F., Hassoun P. (2015). The effects of parity, litter size, physiological state, and milking frequency on the metabolic profile of Lacaune dairy ewes. Domest. Anim. Endocrinol..

